# Adherence with Drug Therapy in Pregnancy

**DOI:** 10.1155/2012/796590

**Published:** 2011-12-26

**Authors:** Doreen Matsui

**Affiliations:** Child Health Research Institute, Departments of Pediatrics and Medicine, Children's Hospital, London Health Sciences Centre, The University of Western Ontario, London, ON, Canada N6C 2V5

## Abstract

Available information suggests that nonadherence with medication is a common problem in pregnant women. Not taking prescribed drugs may have potentially negative consequences as patients may not achieve their therapeutic goal. In addition to the many factors that may influence medication-taking behaviour in the general population, unique challenges are encountered in pregnant women as both maternal health and fetal well-being must be considered. On the one hand, pregnant women may be motivated to keep their underlying disease under control, while, on the other hand, fear and anxiety regarding the potential harmful effects of their medication on their unborn child may result in poor adherence with needed medication. Providing evidence-based information, ideally preconceptually, regarding the effects of their medication during pregnancy may be important in avoiding misperceptions that lead to nonadherence.

## 1. Introduction

Advances in drug therapy have resulted in efficacious treatments being available for many acute and chronic medical conditions; however, it is well recognized that “Drugs don't work in patients who don't take them” [1]. The WHO defines adherence, a term which is often used interchangeably with compliance, as the extent to which a person's behaviour-taking medication, following a diet, and/or executing lifestyle changes, corresponds with agreed recommendations from a health care provider [2]. Unfortunately, nonadherence with medication regimens is not uncommon with the potential negative consequences of failure to achieve the desired treatment goal. Many factors may play a role in whether patients comply with their therapy and pregnancy may present unique challenges as fetal well-being must also be considered in addition to maternal health. This paper will provide a brief overview of medication adherence in general and then will focus on some of the issues related to medication-taking behaviour during pregnancy.

## 2. Overview of Medication Adherence

Nonadherence with drug therapy may take many forms with delayed or omitted doses being the most common errors. Discontinuation of medication administration prior to completion of the course is also common. Adherence is generally measured over a specified period of time and often reported as the percentage of the prescribed doses of medication actually taken by the patient [3]. In a meta-analysis of 569 studies, reported adherence to medical treatment ranged from 4.6% to 100% with a median of 76% and an overall average of 75.2% [4]. Drug compliance may be of particular concern with chronic conditions as therapy is often long term and patients without symptoms may be required to take medication to prevent later complications without any immediate benefits noted. Contrary to what one might think seriousness of the underlying medical condition does not ensure compliance, as has been shown with both cancer and organ transplant therapy [5–9]. Not taking one's medication is not without clinical implications given the relationship between inadequate adherence and unfavourable disease outcome. In a meta-analysis of 21 studies, good adherence with drug therapy was associated with lower mortality compared with poor adherence (odds ratio 0.56, 95% CI 0.50–0.63) [10]. Other consequences of nonadherence may include inappropriate alteration in treatment regimens or dosage adjustments with subsequent toxicity, unnecessary investigations, and increased costs.

Nonadherence with medical therapy is a complex multifaceted problem involving patient and family factors, disease factors, physician factors, and regimen factors [11]. Unfortunately, none of these factors have been found to reliably predict which patients will or will not take their prescribed medication. Various methods are available to assess adherence; however, all have advantages and disadvantages and no universally accepted gold standard exists. The accuracy of self-reporting is often questioned, in particular when the suggestion is that compliance is good. Pill counts may also underestimate adherence as patients “dump” their pills prior to their clinic visit. Measurement of drug levels, available for a limited number of medications, only reflects recent ingestion. Electronic monitors that provide continuous “real-time” measurement can provide information on temporal dosing patterns and allow correlation with breakthrough clinical events. 

It is important to have a high index of suspicion in order to identify early those noncompliant patients who have failed to attain their treatment goal and who may benefit from more targeted support and adherence-enhancing strategies that may include educational and behavioural approaches. Potential barriers to adherence should be identified. No single intervention has been shown to be effective across all patients, conditions, and settings [12]. In a review of interventions for enhancing medication adherence less than one-half of the interventions tested were associated with statistically significant increases in medication adherence and only 29 of 93 interventions reported statistically significant improvements in treatment outcomes. The common theme was more frequent interaction with patients with attention to adherence [13].

## 3. Medication Adherence in Pregnancy

Despite the ample evidence in the literature of the important role of adherence with drug therapy in influencing treatment outcome in the general medical population, there is a relative paucity of studies that have focused specifically on whether pregnant women do or do not take their medication. Much of the research addressing medication compliance during pregnancy has been undertaken in women with HIV infection although there are scattered reports in other medical conditions.

The information available suggests that nonadherence with prescribed drugs is also a problem in the pregnant population. 39% of women who received one or more prescriptions reported noncompliance during pregnancy when interviewed within two weeks after delivery. Reasons included doubts about the use of the drug during pregnancy, expected side effects, disappearance of the complaints for which the drugs were prescribed, or the complaint persisted notwithstanding drug therapy. Approximately 40% of women had had one or more questions about drugs during their pregnancy with safety being the issue that raised most questions [14]. 

Similarly, using data from the North Jutland Prescription Database and from the Danish National Birth Cohort survey, the overall compliance rate with prescription drugs in pregnant women was estimated to be 43% [15]. In the outpatient clinics of an Australian hospital, medication nonadherence was reported by 59.1% of pregnant participants with a chronic health condition. Nonadherence was mainly nonintentional, with forgetting to take medication being the most common reason. In this study, the majority of participants had some concerns about using any medication during pregnancy [16].

### 3.1. Adherence with HIV Medications

Poor adherence with HIV treatment regimens may be a determinant of virologic failure, emergence of drug resistant virus, and disease progression [17]. In addition to treating the mother's underlying disease, antiretroviral treatment in pregnant women also aims to prevent vertical perinatal HIV transmission to the child [18, 19]. Highly active antiretroviral therapy (HAART) has reduced mother-to-child transmission rates to around 1 to 2% in resource-rich countries [20]. Lack of medication adherence in pregnant women with HIV infection may interfere with these goals. Medication nonadherence was a significant factor associated with suboptimal viral suppression at the time of delivery (defined by HIV viral load ≥ 1000 copies) in addition to baseline viral load ≥10,000 copies per milliliter [21].

Studies have shown that HIV-infected pregnant women have greater adherence with antiretroviral drugs than nonpregnant women [22–24]. However, adherence rates during pregnancy are still not optimal and have been reported to be between 43.1% and 80% using various methods of compliance assessment [22–28]. Better compliance with prescribed medications during pregnancy may be related to concern for the baby's health [24, 29]. In the Women and Infant Transmission Study, 90% of women who reported improved adherence with their HIV medications during pregnancy stated that their baby's health was the primary reason [24]. Dosing regimen has also been shown to be important as less than 6 pills per day and up to two doses per day were associated with better adherence [22]. Similarly, pregnant women who were prescribed zidovudine only once or twice daily demonstrated significantly higher adherence than those prescribed this medication three to five times per day [23]. Social support, especially from family members, has a positive influence on medication-taking behaviour [25].

Barriers to good adherence with antiretroviral therapy include being preoccupied with other issues and hectic lifestyles [29] as well as illicit drug use [27, 28]. Untreated depression during pregnancy is also associated with nonadherence to HIV treatment regimens and treatment of depression may improve medication adherence [30]. As poor adherence with antiretroviral drugs during pregnancy may predict nonattendance at infant followup [31], identifying women who are having trouble taking their medication is important.

### 3.2. Adherence with Medications Prescribed for Other Conditions

As in the general population less than perfect adherence with medication taking has been demonstrated in patients with other medical conditions, examples of which will be discussed. 

#### 3.2.1. Epilepsy

Incomplete compliance with anticonvulsant medication was reported by 62.3% (157/252) of pregnant women with epilepsy [32]. Hair analysis was undertaken in 26 pregnant women taking carbamazepine or lamotrigine with four patients (15%) showing declines in drug concentration in the more proximal segments suggesting a change in drug-taking behaviour. Results were interpreted to suggest that these women had discontinued their medication during pregnancy [33].

#### 3.2.2. Asthma

For three categories of asthma severity before pregnancy (intermittent, mild persistent, and moderate/severe), mothers whose medication use fell below the recommended guideline experienced more severe asthma during pregnancy than women using their recommended medication [34]. In an online survey, 39% of women who have been pregnant reported that they had discontinued or reduced their asthma medication during pregnancy, a third having done so without discussion with a physician. Of potential significance in managing these patients, 40% of women indicated that they would be more likely to continue taking their asthma medication during pregnancy if their obstetrician alone recommended it [35]. 40% of pregnant asthmatic subjects reported nonadherence to inhaled corticosteroid medication (ICS). However, after asthma education nonadherence to ICS decreased to 21% [36] which is important to note as lack of appropriate treatment with inhaled corticosteroids is associated with exacerbations of asthma during pregnancy [37]. 

#### 3.2.3. Inflammatory Bowel Disease

Although studies have suggested that exacerbations of inflammatory bowel disease (IBD) during pregnancy may worsen pregnancy outcomes, 84% of female patients with IBD reported concerns that their IBD medications would harm their pregnancy while only 19% reported concerns about the effect of active IBD on pregnancy [38]. In Crohn's disease 67% (37/55) of women reported adherence to medical treatment during pregnancy [39] while in ulcerative colitis 60% (37/62) of women reported adherence [40]. With both conditions, reasons stated for nonadherence included quiescent disease and fear of negative effects on the fetus [39, 40]. 

#### 3.2.4. Nicotine Replacement Therapy

Adherence to nicotine replacement therapy (NRT) is low among pregnant smokers. Fish et al. found that overall only 29% of 104 women used NRT for the recommended 6 weeks and 41% used NRT as directed in the first 48 hours after a quit attempt [41].

## 4. Factors Affecting Medication Adherence in Pregnancy: Perception of Risk

In addition to the multiple factors that may affect medication adherence in the nonpregnant population, there are unique influences that may play a role in pregnant women. On the one hand, pregnant women may be motivated to take their medication for the well-being of their baby, mindful of the potential negative fetal consequences of untreated maternal disease. On the other hand, it has been demonstrated that women may not take their medication due to concerns regarding potential adverse fetal effects [14, 39, 40, 42]. In some cases this fear may be justified based on known teratogenic effects; however, in many cases medications have not been demonstrated to be harmful. It has been shown that pregnant women tend to overestimate the risks associated with drug use during pregnancy. Most women who completed an internet survey were able to correctly identify that the general risk of malformation is ≤5%; however, they overestimated the teratogenic risk associated with many drugs during pregnancy [43].

## 5. Improvement in Medication Adherence in Pregnancy

If women with inadequately controlled disease due to inadequate adherence with drug therapy are identified, they may be targeted for evidence-based counselling to correct their misperceptions, allay their fears, and hopefully improve medication-taking behaviour [43]. In addition, it may be possible to avoid medication nonadherence before it happens by proactively addressing the pregnant woman's concerns about the safety of medications. Ideally, with chronic drug therapy this counselling may occur as part of prepregnancy planning. Survey studies have shown that pregnant women feel they need information about the use of drugs during pregnancy [14, 42]. Media and other sources may provide misleading information provoking anxiety amongst pregnant women [43]. Given the overestimation of risk, the discussion should include, if available, evidence-based information on the effects of the medication's use during pregnancy allowing the pregnant women to make an informed decision as to whether to continue their prescribed therapy. Education should focus on the important role of good medication adherence for the health of both the mother and fetus.

Counselling by teratogen information services may play a role in influencing medication-taking behaviour [43]. After counselling by Motherisk, a Canadian teratogen information service, 61.1% (22/36) of pregnant women who had discontinued their antidepressant or benzodiazepine medication restarted their medication within a few days [44]. Health care workers, such as obstetricians and family physicians, are uniquely positioned to not only monitor adherence but to encourage consultation with drug information services to dispel misconceptions about the risks of medications to the fetus.

A meta-analysis of studies of directly observed therapy of highly active antiretroviral therapy (DOT-HAART) found that DOT-HAART recipients were more likely to achieve undetectable viral load and HAART adherence of greater than or equal to 95% [45]. A similar approach has been suggested for pregnant women. The third trimester of pregnancy may present an opportunity for the use of directly observed HAART to achieve virologic suppression for prevention of mother-to-child transmission of HIV [46]. Using a simulation model, use of DOT in women receiving HAART in 3rd trimester was associated with a relative risk of mother-to-child HIV transmission of 0.39 relative to conventional HAART. It was projected to be highly cost-effective, averting the downstream medical costs associated with pediatric HIV infection [46]. All but one of 17 Latina pregnant and postpartum women positively evaluated a proposed hypothetical modified DOT program [29] suggesting that at least some women would be accepting of this approach.

## 6. Summary

Surprisingly, although it is generally accepted that the maintenance of good health of the pregnant women through the treatment of underlying maternal medical conditions is of potential benefit to the unborn fetus, medication nonadherence is a commonly encountered problem in this population. As in the nonpregnant patient the problem is complex with many factors playing a role; however, additional issues such as the potential effects of the medication on the baby may affect the mother's decision-making process. Ideally, poor compliance with therapy can be avoided through appropriate education of the mother although it is unlikely that this approach will be effective in all cases and solve all problems. More research to provide the evidence-based information regarding the effects of drugs during pregnancy, in order to avoid misperceptions, as well as better knowledge translation is needed. Identification and evaluation of other effective strategies to improve medication adherence in pregnant women who are already not taking their medication will also be important.
